# A Comprehensive Review Comparing Artificial Intelligence and Clinical Diagnostic Approaches for Dry Eye Disease

**DOI:** 10.3390/diagnostics15233071

**Published:** 2025-12-02

**Authors:** Manal El Harti, Said Jai Andaloussi, Ouail Ouchetto

**Affiliations:** Laboratory of Data Engineering and Intelligent Systems, Department of Mathematics and Computer Science, Faculty of Science Ain Chock, Hassan II University of Casablanca, Casablanca 20100, Morocco; said.jaiandaloussi@etu.univh2c.ma (S.J.A.); ouail.ouchetto@etu.univh2c.ma (O.O.)

**Keywords:** dry eye disease, artificial intelligence, machine learning, deep learning, ophthalmologists, clinical tests

## Abstract

This paper provides an overview of artificial intelligence (AI) applications in ophthalmology, with a focus on diagnosing dry eye disease (DED). We aim to synthesize studies that explicitly compare AI-based diagnostic models with clinical tests employed by ophthalmologists, examine results obtained using similar imaging modalities, and identify recurring limitations to propose recommendations for future work. We conducted a systematic literature search following the Preferred Reporting Items for Systematic Reviews and Meta-Analyses (PRISMA) guidelines across four databases: Google Scholar, PubMed, ScienceDirect, and the Cochrane Library. We targeted studies published between 2020 and 2025 and applied predefined inclusion criteria to select 30 original peer-reviewed articles. We then analyzed each study based on the AI models used, development strategies, diagnostic performance, correlation with clinical parameters, and reported limitations. The imaging modalities covered include videokeratography, smartphone-based imaging, tear film interferometry, anterior segment optical coherence tomography, infrared meibography, in vivo confocal microscopy, and slit-lamp photography. Across modalities, deep learning models (e.g., U-shaped Convolutional Network (U-Net), Residual Network (ResNet), Densely Connected Convolutional Network (DenseNet), Generative Adversarial Networks (GANs), transformers) demonstrated promising performance, often matching or surpassing clinical assessments, with reported accuracies ranging from 82% to 99%. However, few studies performed external validations or addressed inter-expert variability. The findings confirm AI’s potential in DED diagnosis, but emphasize gaps in data diversity, clinical use, and reproducibility. It offers practical recommendations for future research to bridge these gaps and support AI deployment in routine eye care.

## 1. Introduction

Dry eye disease is recognized as one of the most frequent ocular disorders; however, prevalence estimates vary considerably across studies. Epidemiological data and findings from the Tear Film & Ocular Surface Society Dry Eye Workshop (TFOS DEWS II report) indicate that between 5% and 50% of individuals in the populations studied are affected by DED [1,2]. Women are disproportionately affected [3], and incidence increases progressively with age [4]. In Africa, the overall prevalence has been estimated at approximately 42% [5]. Contributing factors include prolonged screen exposure, environmental pollution, aging populations, and psychosocial stress [6,7]. DED significantly impairs health-related quality of life (HRQL) and imposes both direct healthcare costs and indirect socioeconomic burdens, including reduced work productivity [8].

DED arises when tear production is insufficient or when the tear film becomes unstable, leading to inadequate ocular lubrication [9]. The tear film comprises three distinct layers—lipid, aqueous, and mucin—each essential for maintaining ocular surface integrity. Dysfunction in any of these layers disrupts tear film stability, initiating or exacerbating DED. Common underlying causes include hormonal fluctuations, inflammation of the eyelid glands, and allergic reactions.

Clinically, DED is categorized into two primary subtypes: aqueous-deficient dry eye (ADDE) and evaporative dry eye (EDE) [10]. ADDE results from reduced aqueous secretion, often associated with aging, Sjögren’s syndrome, thyroid dysfunction, or prolonged medication use. EDE, the more prevalent form, is primarily linked to Meibomian gland dysfunction (MGD), which compromises the lipid layer and accelerates tear evaporation. Additional contributors to EDE include vitamin A deficiency, contact lens wear, and ocular allergies.

Symptoms of DED vary widely and may include dryness, blurred vision, photophobia, eye fatigue, irritation, and, in severe cases, damage to the ocular surface [11]. Common diagnostic indicators include elevated tear osmolarity, diminished tear volume, corneal staining, shortened tear breakup time (TBUT), and reduced tear film stability [12].

According to the TFOS DEWS II and other international consensus reports, the diagnosis of DED requires the presence of ocular symptoms, typically assessed by the Ocular Surface Disease Index (OSDI) with a score of ≥13, in combination with at least one abnormal clinical test result. Common diagnostic benchmarks include a tear film breakup time (TBUT) of <10 s, a Schirmer test value of ≤10 mm/5 min, and a corneal or conjunctival fluorescein staining score of ≥3 [12,13,14].

Diagnosing DED typically involves a battery of tests, broadly classified into invasive and non-invasive approaches. Invasive techniques include TBUT, Schirmer’s test, tear osmolarity measurement, and tear meniscus height evaluation [8]. Non-invasive methods utilize advanced imaging modalities such as in vivo confocal microscopy (IVCM) for corneal nerve analysis [15], meibography for assessing Meibomian gland morphology [16], anterior segment optical coherence tomography (AS-OCT) [17], interferometry (LipiView) [18], infrared thermography [19], and slit-lamp biomicroscopy [20]. According to the TFOS DEWS II report, there is currently no universally accepted gold standard for DED diagnosis [11]. Instead, clinicians typically rely on a combination of tests and symptom-based questionnaires, making the diagnostic process time-consuming and susceptible to inter-observer variability and subjectivity [21].

In recent years, artificial intelligence, particularly machine learning (ML) and deep learning (DL), has shown considerable promise in medical diagnostics, especially within ophthalmology. AI systems have already achieved expert-level performance in detecting conditions such as diabetic retinopathy (DR) [22], and growing interest has focused on extending these capabilities to DED diagnosis [23]. These models can process large volumes of imaging and clinical data with consistency, objectivity, and speed, potentially mitigating the limitations of conventional diagnostic workflows.

Despite these advancements, a significant gap remains between experimental AI model performance and their implementation in routine clinical practice. Much of the current literature lacks direct comparisons with standard clinical diagnostic tools and does not adequately address methodological or practical barriers to adoption. Without rigorous benchmarking against clinical standards and evaluation in real-world conditions, the reliability and translational potential of these AI systems remain uncertain.

To our knowledge, no prior review has systematically compared AI-based diagnostic models against traditional clinical tests across diverse imaging modalities. This review addresses that gap by analyzing 30 peer-reviewed studies published between 2020 and 2025 that explicitly benchmark AI performance against clinician assessments. Unlike previous reviews that focus on general AI trends, we group studies by imaging modality to enable direct comparisons, highlight performance metrics, and critically examine development strategies, data limitations, and clinical applicability. By doing so, we provide a structured roadmap for integrating AI into DED diagnostics and outline key priorities for future model validation and deployment.

Our paper is structured as follows: Section two explores the application of AI in dry eye disease, with a focus on studies employing machine learning and deep learning approaches. Section three outlines our methodology, including the literature search process, inclusion and exclusion criteria, data extraction and categorization procedures, and the assessment of bias and quality of the included studies. Section four presents our results, where we classify the selected articles based on the imaging modalities used. Section five discusses our findings, highlights the identified limitations, and offers recommendations for future research. Section six summarizes the key insights drawn from this study.

## 2. The Application of AI in Dry Eye Disease

Artificial intelligence, a term first introduced in 1956, has gained a significant scope in recent decades due to the availability of large-scale data, advances in computational power and storage, and the development of increasingly sophisticated algorithms. Today, AI is applied across a wide range of sectors, including finance, transportation, and, notably, healthcare. In medicine, AI is being used to address complex diagnostic challenges such as skin cancer classification [24,25], classification of breast cancer histology images [26,27], and tumor detection [28]. Additionally, AI has shown strong potential in ophthalmology, particularly in retinal disease detection [22,29], given the field’s reliance on imaging modalities such as fundus photography, Optical Coherence Tomography (OCT), and meibography, which produce large volumes of data ideal for AI-driven analysis. These systems enhance diagnostic accuracy, reduce inter-observer variability, and support clinical decision-making.

### 2.1. ML for Dry Eye Disease Diagnosis

Machine learning has emerged as a powerful tool in dry eye disease diagnosis by analyzing large sets of imaging and clinical data to uncover complex patterns beyond human perception. Nam et al. [30] developed an explanatory model for DED using national health and nutrition survey data, applying machine learning and network-based factor analysis to identify systemic and lifestyle risk factors associated with the disease. Similarly, Sheshkal et al. [31] employed metabolomics data and compared several ML algorithms—including Logistic Regression (LR), Random Forest (RF), and XGBoost—to distinguish DED patients from healthy subjects, achieving an Area Under Curve (AUC) of 0.83. In addition, Jarada et al. [32] trained supervised ML models on real-world clinical parameters such as the Schirmer test, tear break-up time, and corneal staining scores, enabling automatic prediction of DED severity. Furthermore, Patel et al. [33] demonstrated that infrared thermography images combined with ML classifiers such as Support Vector Machines (SVMs) and k-Nearest Neighbors (k-NN) can effectively differentiate DED patients from healthy controls, reaching an accuracy of up to 90%. Additionally, Kopro et al. [34] developed a machine learning method to assess Meibomian gland quality from meibography images, demonstrating clinical utility in evaluating gland dysfunction—a key factor in evaporative DED. Collectively, these studies underscore the versatility of traditional machine learning techniques in integrating multimodal data—from metabolic and thermal imaging to clinical measurements—to enhance diagnostic precision and support clinical decision-making in DED management.

### 2.2. DL for Dry Eye Disease Diagnosis

Deep learning has revolutionized retinal disease diagnosis by enabling automated feature extraction directly from raw medical images, removing the need for manual feature engineering. Among deep learning architectures, convolutional neural networks (CNNs) have proven especially effective at recognizing complex retinal patterns [35]. Su et al. [23] proposed a CNN-based deep learning model that automatically measured tear film break-up time from video sequences, achieving a mean absolute error (MAE) of 0.49 s compared to expert annotations. Extending DL to the corneal domain, Elsawy et al. [36] introduced a multi-disease neural network built on the ResNet-50 architecture [37], capable of diagnosing various corneal conditions, including DED, from anterior segment images. Their model achieved an AUC of 0.983 for DED classification, indicating excellent diagnostic accuracy. Furthermore, Ren et al. [38] compared a deep learning-assisted blink analysis system with a commercial interferometer (Lipiview), confirming that CNN-based systems can reliably quantify lipid layer thickness and blink dynamics in DED patients.

## 3. Methodology

**Figure 1:**
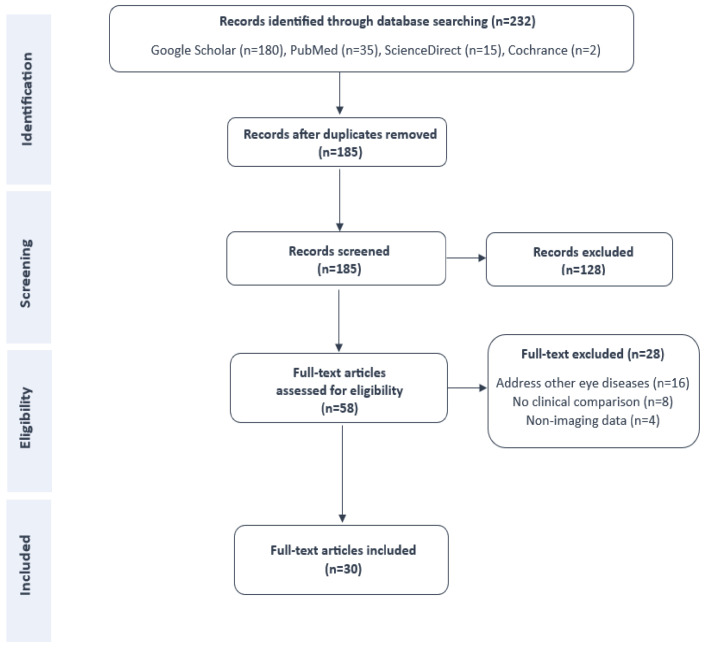
PRISMA flow of the selection process.

To assess the reliability of AI in diagnosing dry eye disease and its potential for clinical integration, we conducted a systematic literature search covering articles published between 2020 and 30 April 2025. We specifically focused on studies comparing AI-based diagnostic performance with ophthalmologists’ assessments or correlating AI results with established clinical parameters. The search covered four major open-access academic databases: Google Scholar, a broad search engine indexing a wide range of scholarly literature across disciplines. ScienceDirect, a database specializing in scientific and technical research, particularly from Elsevier journals. PubMed, a trusted source of biomedical and clinical literature, maintained by the United States National Library of Medicine. Cochrane Library, known for its systematic reviews and high-quality evidence in healthcare and clinical research. These platforms provide free access to academic publications and do not require institutional subscriptions. We limited our search to English-language articles. The search strategy used keywords applied to titles, abstracts, and metadata. The review process adhered to the PRISMA guidelines [39]. A PRISMA flowchart summarizing the article selection process is shown in Figure 1.

### 3.1. Search Strategies

Our literature search employed a targeted set of keywords combining terms related to dry eye disease, clinical diagnosis, and artificial intelligence. Specifically, we used (“artificial intelligence” OR “machine learning” OR “deep learning”) AND “dry eye disease” AND (“ophthalmologists” OR “clinical”). After retrieving the initial set of publications, we removed duplicate entries across databases using a combination of automated tools and manual screening. We then assessed the remaining articles individually according to predefined inclusion criteria.

### 3.2. Inclusion and Exclusion Criteria

To be qualified for inclusion, studies were required to meet the following conditions:

- Language and access: Full-text versions of the articles must be available in English.
- Clinical focus: The study must specifically address the use of artificial intelligence, including machine learning or deep learning methods, for the diagnosis or classification of dry eye disease.
- Descriptive completeness: Eligible studies were expected to provide the following:
  - The year of publication.
  - Details about the datasets used, including data acquisition and processing methods.
  - A clear description of the study approach and identification of the employed learning model.
  - An evaluation of model performance, including metrics such as accuracy, AUC, sensitivity, or specificity.
  - Analysis of the model’s correlation with clinical parameters (e.g., TBUT, Schirmer’s, staining, meibography).
  - A comparison of automatic DED diagnosis versus expert or clinician assessment.
  - A concise summary of study objectives and primary outcomes.

Studies were eliminated if they matched any of the following criteria:

- The article was a review, editorial, or commentary rather than original research.
- The study focused on ocular anomalies unrelated to DED.
- The investigation did not include a performance comparison between AI-based and human diagnosis, or lacked sufficient methodological transparency for evaluation.
- Studies that relied on non-imaging modalities or questionnaire-based methods for DED diagnosis.

### 3.3. Data Extraction and Categorization

After screening studies for inclusion and full-text eligibility, we retained those that compared AI-based DED diagnosis with clinician assessments. From each included study, we extracted a predefined set of variables and organized the findings into structured tables, placing them within the corresponding imaging modality section to enhance readability. The key data elements included the following:

(1) The year of publication: We recorded the publication year of each study to highlight the temporal evolution of artificial intelligence applications in DED diagnosis.
(2) Study Purpose: We identified the objective or diagnostic task in each study by analyzing its abstract and introduction. This allowed us to classify the studies based on their focus, such as gland segmentation, tear film assessment, or subtype classification.
(3) Learning Model: The reviewed studies employed a variety of artificial intelligence models, including machine learning algorithms (e.g., SVM, RF) and deep learning architectures (e.g., CNN, U-Net, GANs). We examined each model based on its architecture and configuration.
(4) Data Source: We extracted information about each dataset, including the image modality (e.g., meibography, OCT, IVCM), sample size, training/test splits, and data acquisition tools. We also noted the dataset’s quality, diversity, and annotation methods, given their critical impact on model performance.
(5) Development Strategy: We examined how each study structured its development pipeline, including preprocessing, training, validation, and testing procedures. We paid special attention to practices that enhance reproducibility, such as cross-validation, data augmentation, and external validation.
(6) Correlation with Experts: We recorded whether the AI model’s performance was explicitly compared to clinical assessments by ophthalmologists or correlated with diagnostic parameters such as TBUT, Schirmer’s test, staining, or meiboscore.
(7) Results: We reviewed each study’s diagnostic performance, focusing on reported metrics such as accuracy, sensitivity, specificity, AUC, and inter-expert agreement. We also emphasized whether the AI system outperformed or complemented human diagnosis.
(8) Limitations: We highlighted reported limitations such as restricted dataset size, lack of external validation, overfitting risk, or limited translation into clinical practice, which point to opportunities for future work.

It is important to note that the reference standards used across the included studies were heterogeneous. In most cases, AI models were trained and validated against clinician-provided labels or results from individual diagnostic tests such as tear film breakup time, Schirmer test, meibography, or corneal staining. Only a limited number of studies explicitly reported adherence to the comprehensive TFOS DEWS II diagnostic algorithm, which combines symptom assessment with at least one abnormal objective test. This variability in ground-truth definition should be considered when interpreting model performance, as it may contribute to discrepancies in reported accuracy and generalizability across studies.

### 3.4. Assessment of Bias and Quality of Included Studies

To evaluate the methodological quality and potential risk of bias among the included studies, we adopted criteria adapted from the QUADAS-2 framework [40]. This tool assesses diagnostic accuracy studies across four key domains: patient selection, index test, reference standard, and flow and timing. In this review, each study was qualitatively analyzed with respect to dataset representativeness, clarity of ground-truth definition, validation strategy, and transparency in performance reporting. The assessment ensured that potential sources of bias were identified and considered when interpreting the results.

## 4. Results

Following the initial database search, a total of 232 potentially relevant records were retrieved: 180 from Google Scholar, 15 from ScienceDirect, 35 from PubMed, and 2 from the Cochrane Library. After removing duplicates, 185 unique articles remained. These were screened for eligibility based on titles and abstracts, followed by a full-text review using predefined inclusion and exclusion criteria.

After manual assessment, we selected 30 studies that met all inclusion criteria, specifically those comparing AI-based models with expert ophthalmologists or established clinical parameters for diagnosing dry eye disease. These studies were included in the final comprehensive review and categorized according to the imaging modality employed: videokeratography (n = 1), smartphone-based imaging (n = 2), tear film interferometry (n = 3), anterior segment optical coherence tomography (n = 1), infrared meibography (n = 9), in vivo confocal microscopy (n = 6), and slit-lamp photography (n = 8). For each study, we summarize the diagnostic objective, learning model, dataset characteristics, development methodology, performance metrics, correlation with expert assessments, and key limitations.

### 4.1. Videokeratography

Videokeratography, a non-invasive technique for mapping corneal curvature, provides indirect insights into tear film stability by capturing topographic changes linked to fluorescein breakup patterns. Its value as a dynamic diagnostic tool for dry eye disease lies in its ability to record temporal alterations in tear film integrity across the ocular surface.

In the sole study identified using this modality, Yokoi et al. [41] developed a deep learning model to classify DED subtypes from fluorescein breakup video sequences. Unlike static image-based methods, this approach analyzed both spatial and temporal information, addressing the clinical need to evaluate dynamic tear film behavior. The dataset included 243 eyes, from Kyoto Prefectural University of Medicine, classified according to the tear film-oriented diagnosis (TFOD) criteria, expanded through data augmentation to balance classes. The model achieved an overall accuracy of 78.4%, with higher performance for severe aqueous-deficient DED (92.3%), reflecting its sensitivity to more distinct pattern variations compared to milder forms. Table 1 summarizes the potential and limitations of videokeratography-based AI models for DED diagnosis.

### 4.2. Smartphone-Based Imaging

Smartphone-based ocular imaging has gained traction as an accessible, low-cost alternative for anterior segment evaluation in dry eye disease. Leveraging the prevalence and improving the optical quality of mobile devices, recent AI frameworks have explored their integration into semi-automated or fully automated DED diagnostic tools. Two studies addressed distinct clinical targets using different smartphone platforms, pipelines, and output modalities, highlighting the modality’s versatility.

In [42], Nejat et al. proposed a pipeline combining U-Net, MobileNetV2 [43], and Laplacian Pyramid Networks to estimate tear meniscus height from 1021 smartphone-acquired images. The model achieved an accuracy of 95.39%, demonstrating precise anatomical segmentation. However, the results were based solely on internal data, and no correlation with clinical scores was reported, limiting clinical interpretability and external validity.

In contrast, Rodriguez et al. [44] focused on grading conjunctival hyperemia using 29,640 images collected from clinical trials. A DeepLabV3 model [45] was employed to segment the conjunctiva and extract redness and vascularity features, which were subsequently analyzed using statistical models. The system achieved 93.0% agreement within one grade of expert assessments, outperforming inter-rater human consistency, which stood at 85.8%.

While Nejat et al. [42] focused on structural biomarkers for potential home-based assessment, Rodriguez et al. [44] emphasized scalable, subjective grading aligned with clinical trial settings. Both studies demonstrated strong model performance; however, neither included external validation or real-world usability evaluations, limiting their immediate clinical applicability. The details of these studies are summarized in Table 2.

### 4.3. Tear Film Interferometry

Tear film interferometry has emerged as a valuable tool for analyzing blinking behavior and tear meniscus characteristics—both essential for the accurate assessment of DED. Recent studies have applied deep learning to automate its interpretation, enhancing objectivity and reducing examiner-dependent variability.

A U-Net model was used by Zheng et al. [46] to analyze blink completeness from OCULUS Keratograph 5M video sequences, achieving high segmentation performance (sensitivity: 0.994). Incomplete blinking was found to be significantly more frequent in DED patients and showed a strong correlation with clinical metrics such as tear meniscus height (TMH) and OSDI, underscoring its diagnostic relevance.

In comparison, Zhang et al. [47] employed a Mask R-CNN [48] with a ResNet-101 backbone to segment the tear meniscus in static images, achieving strong agreement with manual measurements (intersection over union (IoU) = 0.928, R^2^ = 0.92) and excellent external validation (AUC = 0.975).

Similarly, Wan et al. [49] proposed a DeepLabv3-based model with a hybrid architecture, reaching an r^2^ of 0.94, despite a smaller dataset. Their inclusion of corneal ring segmentation improved spatial accuracy.

Overall, these studies demonstrate complementary deep learning approaches to tear film analysis using interferometry. While blink completeness analysis offers dynamic, functional insights into evaporative DED mechanisms, TMH-based models provide structural, quantitative assessments of tear volume. The consistent combination of high segmentation accuracy and strong clinical correlation across diverse model architectures highlights the maturity and adaptability of deep learning in DED diagnosis. Nevertheless, differences in sample size, input modality (video vs. static images), and evaluation protocols indicate that model generalizability and clinical applicability should be interpreted with caution and in context.

The details of these three studies are summarized in Table 3.

### 4.4. Optical Coherence Tomography

Optical coherence tomography provides high-resolution cross-sectional imaging of anterior segment structures, enabling a quantitative analysis of parameters including tear meniscus height, corneal epithelium, and tear film layers—parameters relevant to dry eye disease.

A VGG19-based deep learning model was developed by Collin et al. [50] to classify DED using 27,180 AS-OCT images from 151 eyes of 91 patients. Low-quality images were excluded to ensure reliability. Model performance was evaluated using accuracy and cross-entropy, achieving an overall accuracy of 84.62%. Feature visualization techniques revealed that the model primarily attended to anatomically relevant layers, including the tear film and corneal epithelium.

Comparative analysis using the Mann–Whitney U-test and McNemar’s test demonstrated that, although the DL model outperformed individual clinical tests—such as TBUT, OSDI, Schirmer’s test, and corneal staining—its diagnostic accuracy was not statistically superior to the combined clinical assessments routinely employed by ophthalmologists. These findings suggest that AI-based OCT analysis holds promise as a supportive diagnostic tool, providing an automated, high-throughput evaluation comparable to expert clinical judgment.

The main findings of this study are summarized in Table 4.

### 4.5. Infrared Meibography

Infrared meibography remains the most commonly explored imaging modality in AI-assisted DED diagnostics, particularly for assessing Meibomian gland dysfunction. Across nine studies, researchers employed deep learning models—primarily CNN and U-Net variants—to automate gland segmentation, quantify morphological features, and classify disease severity.

Dataset sizes varied considerably, ranging from 60 to 143,476 images. The most extensive effort by Scales et al. [51] introduced a modular pipeline of three CNNs trained on 143,476 LipiScan images, collected from multiple sites across North America, achieving high agreement with expert annotations (AUC = 0.93; κ = 0.71). In contrast, Dai et al. [52] addressed the challenge of limited data by applying data augmentation to only 60 images from the Oculus Keratograph 5M, enabling a mini U-Net to reach IoU = 0.91 and show significant correlations with clinical scores such as TBUT and OSDI.

Model architecture and preprocessing also influenced performance. Wang et al. [53] combined ResNet18 and U-Net within an adversarial training (VAE-GAN) [54], obtaining high specificity (>96%) but moderate sensitivity (75.1%), highlighting the model’s robustness yet vulnerability to eyelash interference. Similarly, Yu et al. [55] used Mask R-CNN on 1878 annotated images, leveraging transfer learning to achieve faster-than-clinician processing (0.48 s/image) with an r = 0.976 correlation in MG quantification.

Other models focused on segmentation refinement. Ref. [56] pretrained Inception-ResNet on chest X-rays before fine-tuning U-Net on 728 Mebiomian gland (MG) images, acquired using an Oculus Keratograph 5M device at the Eye Hospital, Department of Ophthalmology, University Hospital Cologne, Germany, achieving 100% repeatability and 84% segmentation accuracy. Meanwhile, Saha et al. [57] implemented a multi-task ResNet34 pipeline integrated with GAN-based reflection correction and meiboscore classification. Despite an internal IoU of 67.63%, external test accuracy dropped to 59.17%, suggesting susceptibility to inter-expert variability.

Two studies explored predictive tasks beyond segmentation. Graham et al. [58] used a traditional supervised pipeline to link MG morphology with clinical signs across 562 images, predicting eyelid notching (95.9%) and aqueous deficiency (85.2%) from gland features alone. Rajan et al. [59] went further by introducing a hybrid model combining UNet++ [60], feature selection, and an optimized sequence-to-sequence classifier trained on 1000 LipiView II images, outperforming all tested baselines with 96.34% classification accuracy.

Finally, Sahana et al. [61] proposed CNN19 trained on 800 multi-device images, using novel elastic and perturbation augmentation techniques. The model closely matched ground-truth metrics despite a limited training set.

Across these studies, U-Net variants remained the preferred choice for segmentation, while hybrid and multi-task models achieved enhanced interpretability and generalization. However, only a few works included external validation or addressed clinician-level inter-rater variability—both critical factors for real-world deployment.

The key findings of these nine studies are summarized in Table 5.

### 4.6. In Vivo Confocal Microscopy

In vivo confocal microscopy provides high-resolution imaging of corneal nerves and Meibomian glands, making it a valuable modality for detecting neuropathic alterations and assessing Meibomian gland dysfunction in DED. Six AI-based studies have leveraged IVCM for tasks such as segmentation, classification, and feature quantification, employing varied model architectures and targeting different anatomical structures.

For Meibomian gland dysfunction classification, Yi et al. [62] applied a ResNet34 model to 24,919 images from diverse sources, reaching 86.1% accuracy—comparable to expert diagnoses. Using 64 convolutional kernels, the model demonstrated that even conventional CNNs can perform robustly at scale. In a more detailed classification task, Zhang et al. [63] trained three DenseNet variants on 8311 images to distinguish obstructive from atrophic MGD, with DenseNet169 achieving up to 99% accuracy—substantially surpassing the 91% accuracy achieved by ophthalmologists.

For corneal nerve analysis, several models addressed segmentation and morphological quantification. Wei et al. [64] introduced corneal nerve segmentation (CNS-Net), a specialized DL model trained on 691 annotated IVCM images, collected at Peking University Third Hospital, achieving AUC = 0.96. Similarly, Yildiz et al. [65] compared a GAN-based model to U-Net on 505 images, reporting higher AUC (0.944 vs. 0.893) and greater robustness to image noise. Under salt-and-pepper and speckle noise, the GAN maintained diagnostic precision, while U-Net’s accuracy dropped notably (0.818 vs. 0.878).

Two studies extended IVCM applications beyond classification. Baikai et al. [66] quantified corneal nerve tortuosity using local-phase enhancement and infinite perimeter active contour with hybrid region information (IPACHI) segmentation on 1501 images. Their KNN-based classifier revealed that tortuosity (tagg) correlated positively with OSDI scores (r = 0.418) and negatively with TBUT (*r* = −0.398), reinforcing the clinical relevance of nerve morphology. Meanwhile, Setu et al. [67] proposed a dual-task deep learning pipeline combining U-Net and Mask R-CNN (ResNet101 backbone) to segment corneal nerve fibers (CNFs) and dendritic cells (DCs) simultaneously. Trained on 1219 CNF and 754 DC images, acquired at the University Hospital Cologne, the system achieved high precision (89.4%), and interclass correlation coefficients exceeded 0.95 in several categories.

Overall, DenseNet- and ResNet-based classifiers demonstrated high accuracy in large-scale MGD classification, while U-Net- and GAN-based methods excelled in detailed nerve segmentation. Studies such as [66,67] advanced IVCM analysis by quantifying disease-specific morphological markers, offering more nuanced and clinically relevant assessments. However, the limited inclusion of robustness testing and external validation across studies underscores the need for a greater emphasis on clinical generalizability and inter-center reproducibility.

The key findings of the mentioned studies are summarized in Table 6.

### 4.7. Slit-Lamp Photography

Slit-lamp photography offers a non-invasive method to assess ocular surface integrity, particularly via fluorescein staining and tear film breakup. Recent AI-driven studies have leveraged this modality for both automated grading and disease classification, using either static images or dynamic video inputs. Eight studies explored diverse architectures, dataset scales, and grading frameworks to improve diagnostic accuracy and reproducibility in dry eye evaluation.

Early studies favored traditional machine learning approaches. Feng et al. [68] extracted topological, morphological, and textural features from 382 slit-lamp images collected from the retrospective dataset of Beijing Tongren Hospital (Capital Medical University). Moreover, they used SVMs to classify dry eye severity. Their model reached 82.67% accuracy and AUC = 96.59%, outperforming subjective grading scales. Similarly, Kourukmas et al. [69] applied an ImageJ-based rule system on 50 corneal-stained images, achieving high correlation with true Oxford grades (Sr = 0.91) and perfect consistency across grading sessions (K = 1.0), in contrast to the moderate inter-rater agreement among human evaluators (K = 0.426).

More recent work adopted deep learning to enhance scalability and generalization. Kim et al. [70] developed a three-step system incorporating U-Net for segmentation, VGG16 for punctate erosion classification, and a blob detector to estimate National Eye Institute (NEI) grades from 1400 images, plus 94 external validation samples. The pipeline achieved AUC = 0.97, and strong correlation with expert grading (r = 0.868 − 0.863), also tracking disease progression in 88% of longitudinal cases. Likewise, Yuan et al. [71] proposed a fine-grained knowledge distillation corneal staining score (FKD-CSS), a dual-decoder framework trained on 1471 clinical trial images and tested on 2376 images from 23 hospitals, achieving AUC = 0.883 and surpassing senior ophthalmologists in grading accuracy. Its fine-grained attention maps enhanced interpretability, while robust performance across multiple ocular surface conditions suggested strong generalization.

Two studies focused on video-based tear film assessment. Shimizu et al. [72] used a Swin Transformer, pretrained on ImageNet-22k, to analyze 16,440 video frames from the Smart Eye Camera, achieving TFBUT detection accuracy = 78.9%. The model’s predictions correlated moderately with clinical scores (r = 0.791), and Grad-CAM confirmed spatial relevance. Similarly, El barche et al. [73] introduced a Dual-Task Siamese Network trained on 67 ION Imaging videos, where InceptionV3 achieved AUC = 0.870 and r = 0.81 against clinician-assessed TFBUT, supported by temporal smoothing and augmentation strategies.

Su et al. [74] proposed CNN-SPK, a lightweight 6-layer CNN trained on 101 images, estimating punctate dot coverage with 97% accuracy and a strong Pearson correlation (r = 0.85) to clinical grading. The model was simple yet effective, even without preprocessing.

Finally, Li et al. [75] introduced a dual-mode system combining red, green, and blue (RGB) slit-lamp and infrared meibography images to classify ocular surface disease (OSD) subtypes and evaluate Meibomian gland morphology. Using YOLOv5 for classification and Specialized U-Net (SU-Net) for segmentation, the model was trained on 3640 internally collected images from the Lo Fong Shiu Po Eye Centre, along with the publicly available Sclera Blood Vessels, Periocular, and Iris (SBVPI) dataset, comprising 1840 external eye images from 55 subjects. The system achieved 98.7% classification accuracy across DED, conjunctivitis, and Subconjunctival Hemorrhage (SCH), with 88.1% accuracy for MG-related features.

Collectively, these studies demonstrate a shift from manual or handcrafted-feature grading to deep learning systems capable of objective, reproducible, and high-performance DED assessment. Transformer and Siamese architectures excel in dynamic video interpretation, while U-Net variants and interpretable DL frameworks (e.g., FKD-CSS) show promise in large-scale, multi-center validation. However, only a few works evaluated external reproducibility or addressed inter-clinician variability—factors critical for real-world clinical deployment.

Table 7 summarizes the main findings of these eight studies.

## 5. Discussion

### 5.1. Comparative Analysis: AI Versus Clinical Diagnostic Methods

While AI systems have shown substantial promise in automating the diagnosis of DED, a careful comparison with traditional clinical methods is necessary to evaluate their practical applicability. Among the 30 studies reviewed, deep learning models reported accuracies ranging from 82% to 99%, frequently matching or exceeding clinician performance on specific diagnostic tasks.

For instance, the dual-modality system combining U-Net and YOLOv5 applied to slit-lamp images, by Li et al. [75], achieved an accuracy of 98.7% and an F1 score of 0.980, outperforming typical clinician-based assessments, which generally range between 80% and 90% depending on the test and observer expertise. These findings underscore the strong promise of meibography as the leading modality, particularly when enhanced with slit-lamp input. This combination allows for a comprehensive visualization of both the Meibomian glands and external ocular surface, making it especially effective in identifying evaporative DED—the most prevalent subtype. Similarly, a ResNet-based approach for Meibomian gland segmentation and tarsus evaluation by Wang et al. [53] reached 98.5% accuracy with AUC values exceeding 0.98, while a DenseNet169 classifier applied to VCMI images by Zhang et al. [63] achieved 99% accuracy in classifying advanced MGD cases. By contrast, conventional tests such as Schirmer’s or TBUT often exhibit limited sensitivity and reproducibility, particularly in early-stage or borderline cases.

In terms of processing speed, AI systems present a marked advantage. Once trained, most models can analyze an image in under 1–2 s, enabling real-time or near-real-time diagnostic support. In comparison, a full clinical assessment—including Schirmer’s test, TBUT, staining, and image interpretation—typically requires 10 to 20 min per patient and is highly operator-dependent.

AI systems also offer potential benefits in cost-effectiveness and accessibility. Several studies deployed models on smartphone-based or portable platforms [42], significantly reducing diagnostic costs by eliminating the need for high-end equipment such as OCT or interferometers. This opens opportunities for implementation in remote or resource-limited settings. For example, a U-Net + MobileNetV2 model used for tear meniscus height estimation via smartphone achieved performance comparable to OCT, with a strong correlation (r = 0.94), while maintaining minimal deployment costs.

Nevertheless, traditional clinical methods retain advantages in certain scenarios. Experienced ophthalmologists can detect subtle clinical indicators, incorporate patient history, and account for comorbidities—factors that are often beyond the scope of current AI models. Moreover, tests such as tear osmolarity measurements and slit-lamp evaluations are well-regulated and widely validated, whereas approximately 70% of the studies reviewed lacked external validation and only a few assessed cross-device or cross-population generalizability.

In conclusion, AI-based systems offer significant improvements in speed, cost, and consistency, particularly for screening, monitoring, and supporting decision-making in high-throughput or underserved settings. However, clinical expertise remains indispensable for managing complex cases and ensuring diagnostic interpretability until AI systems are more extensively validated and integrated into regulatory frameworks.

### 5.2. Limitations and Challenges

This review highlights the significant potential of artificial intelligence in advancing dry eye disease diagnosis; however, it also reveals recurring methodological limitations and unmet requirements that currently hinder widespread clinical adoption. Across the 30 studies analyzed, the following key challenges emerged:

- Restricted scope and variability of training datasets: Most models were trained on monocentric datasets, typically using images from a single eye per patient and acquired using one imaging device or modality. This limited heterogeneity constraints’ generalizability. For example, Nejat et al. [42] developed a smartphone-based tear meniscus height measurement model using only images from iPhoneand Samsung devices. While the model achieved a high Dice coefficient (98.68%), it lacked cross-device validation. Similarly, Wan et al. [49] reported a strong correlation with expert annotations ($r^{2}\;=\;0.94$) using Keratograph 5M images, yet the dataset consisted of only 305 samples from a single site. Even larger studies, such as the one by Graham et al. [58], which used 562 images from 363 subjects, applied exclusion criteria (e.g., prior surgery, inflammation), which may limit the applicability of results to the broader DED population. Moreover, few studies have evaluated model performance across different ethnic groups, imaging devices, or clinical settings, which limits confidence in their robustness for diverse real-world applications. Future work should prioritize multi-center, multi-ethnic, and cross-device datasets to ensure equitable and generalizable AI systems for DED diagnosis.
- Lack of external and cross-device validation: While many models demonstrated expert-level performance on internal test sets, their generalizability across clinical settings remains largely unverified. For instance, Feng et al. [68] trained SVM classifiers on 382 slit-lamp images from a single institution and reported AUCs up to 96.59%. Likewise, Saha et al. [57]’s meiboscore model performed well on internal data (73.01% accuracy) but showed a marked drop on external datasets (59.17%), underscoring the need for multi-center datasets and domain adaptation strategies.
  Although most studies in this review reported promising results, a large portion relied solely on internal validation without testing on external or real-world datasets. This limitation reduces the generalizability and clinical applicability of their findings, as models may overfit to specific imaging conditions or patient populations. Only a few studies incorporated external testing or multi-center data, highlighting the need for standardized validation protocols and real-world evaluation before clinical deployment. Recent works [8,72] have emphasized that external validation across diverse cohorts is essential to ensure robust and trustworthy AI tools for dry eye disease diagnosis.
- Annotation variability and quality of ground-truth labels: Few studies evaluated inter-rater reliability or the robustness of ground-truth annotations. Saha et al. [57] addressed this limitation by conducting six rounds of meiboscore labeling and assessing consistency using Cohen’s kappa and Bland–Altman analysis. Despite these efforts, 59 images were excluded due to artifacts or severe gland dropout. Similarly, Setu et al. [67] noted that even advanced DL models struggled with image artifacts such as poor contrast, defocus, and specular reflections—challenges not fully resolved by classical preprocessing methods.
- Lack of symptom-based or functional integration: Many studies focused exclusively on morphological features (e.g., gland tortuosity, nerve length), with limited inclusion of patient-reported outcomes or functional clinical tests such as TBUT or OSDI. Graham et al. [58] notably integrated imaging features with validated questionnaires and clinical signs, achieving predictive accuracy up to 99%. However, even in this case, the model lacked interpretability regarding feature contribution, highlighting the growing need for explainable AI (XAI) approaches.
- Impact of TFOS DEWS II Criteria Implementation Although the TFOS DEWS II framework establishes standardized diagnostic thresholds that combine subjective symptoms such as an OSDI score of ≥13 with at least one abnormal objective test, this review found that adherence to these criteria varied widely across the analyzed studies. Several works classified DED based on single-parameter evaluations such as meiboscore [57], tear meniscus height [42,47,49], or TBUT [50,52,66], while others relied solely on clinician judgment without specifying the use of TFOS DEWS II benchmarks. This heterogeneity in diagnostic definitions introduces label inconsistency, potentially biasing AI model performance and complicating cross-study comparisons. Consequently, high reported accuracy in some models may partly reflect dataset-specific labeling strategies rather than true diagnostic generalizability.
- Limited explainability and clinical interpretability: Most models operated as “black boxes,” providing limited insights into their internal decision-making processes—an issue that can undermine clinical trust, regulatory approval, and adoption. Only a few studies, such as the one by Yuan et al. [71], incorporated interpretability tools such as heatmaps and lesion-aware visualization to highlight key diagnostic regions. However, the systematic evaluation of model explainability remains scarce. Broader implementation of explainable artificial intelligence (XAI) techniques—such as saliency mapping [76], Grad-CAM visualization [77], and attention mechanisms [78]—is essential to bridge the gap between high algorithmic performance and clinical confidence, ensuring transparency, reproducibility, and safer AI integration in ophthalmic practice.

Despite these challenges, several promising directions are emerging. Li et al. [75]’s dual-mode RGB and infrared imaging system demonstrates strong multi-class classification performance, while Nejat et al. [42]’s smartphone-based pipeline offers a scalable pathway for at-home screening. Likewise, the integrative approach presented by Graham et al. [58], which combines clinical symptoms with imaging features, suggests a viable framework for aligning AI-based tools with real-world diagnostic workflows.

### 5.3. Recommendations and Future Perspectives

All studies included in this review were published within the recent five years, reflecting the rapid and recent expansion of artificial intelligence applications in medical imaging—particularly in diagnosing ocular surface diseases such as dry eye disease. Despite encouraging progress, several methodological gaps remain, which must be addressed to enable broader clinical translation of AI-based systems.

- Establish centralized, multimodal, publicly available datasets: None of the reviewed studies utilized open-access datasets. Most relied on data collected at single institutions under controlled conditions. For instance, Setu et al. [67] and Saha et al. [57] employed internal meibography datasets, while Nejat et al. [42] trained their model using images from only two smartphone types. Such limited scope restricts model generalizability across devices and populations. Future initiatives should prioritize the creation of standardized, multi-center datasets incorporating various imaging modalities (e.g., RGB, infrared, OCT), along with corresponding clinical metrics and symptom-based data.
  Precedents from other medical domains underscore the feasibility and benefits of this approach. The Brain Tumor Segmentation Challenge (BraTS) 2018 dataset [79], for example, aggregated multimodal Magnetic Resonance Imaging (MRI) scans from 19 institutions, while the Ophthalmologie Diabète Télémédecine (OPHDIAT) telemedicine network [80,81] facilitated diabetic retinopathy screening across 16 centers. Similar efforts in DED could greatly enhance reproducibility, support benchmarking, and improve model robustness.
- Utilize data augmentation to address dataset limitations: Data augmentation remains an effective strategy for improving data diversity and mitigating class imbalance. Common techniques—such as flipping, rotation, intensity perturbation, and elastic deformation—have consistently enhanced model performance in DED applications. For example, Feng et al. [68] and Zheng et al. [46] employed DA to improve segmentation outcomes, while Nejat et al. [42]’s preprocessing and fusion pipeline increased TMH estimation accuracy to 95.39%. In other domains, DA has been shown to improve prostate segmentation accuracy by 12–47% and to strengthen brain tumor classification using class-balanced learning strategies [82,83].
- Prioritize model interpretability and clinical transparency: Explainable AI should be embedded into model development from the outset. Beyond high performance, clinical deployment demands that models offer interpretable outputs. Techniques such as class activation mapping (CAM), attention mechanisms, and symbolic reasoning can enhance transparency and foster trust. For example, Jiang et al. [84] used CAMs to highlight diabetic retinopathy features, while Gwenolé et al. [22] introduced “ExplAIn,” an interpretable AI framework that visualized relevant image regions to support physician decision-making.

Addressing these critical challenges—namely, dataset quality, external validation, interpretability, and multimodal integration—will be essential for advancing AI systems from experimental tools to clinically dependable assistants. The future of AI in DED diagnosis lies in developing robust, transparent, and equitable models that are closely aligned with real-world clinical workflows.

### 5.4. Theoretical Implications

This review extends the theoretical foundation of diagnostic intelligence by demonstrating how artificial intelligence models emulate and enhance clinical reasoning in ophthalmology. It provides a conceptual framework linking data-driven pattern recognition with traditional pathophysiological assessment, bridging the gap between algorithmic learning and clinician-based judgment. The findings contribute to the theoretical discourse on hybrid intelligence systems, emphasizing their role in augmenting, rather than replacing, human expertise.

## 6. Conclusions

This review demonstrates that artificial intelligence, particularly deep learning applied to ocular imaging, holds significant promise for enhancing the diagnosis of DED. Across diverse imaging modalities—including infrared meibography, optical coherence tomography, and tear film interferometry—AI systems consistently achieved high diagnostic accuracy, often matching or exceeding traditional clinical tests such as TBUT, OSDI, and Schirmer’s test. Among these, infrared meibography stands out as the most clinically advanced, owing to its direct visualization of Meibomian gland morphology and strong correlation with DED signs.

Despite these promising results, most AI-based models remain confined to experimental contexts. A significant barrier to clinical translation lies in their development on limited or single-center datasets, with little external validation, minimal prospective testing, and no integration into real-world clinical workflows. Additionally, factors such as annotation variability, hardware dependency, and the absence of standardized evaluation protocols hinder broader adoption.

Bridging this research–practice divide will require coordinated efforts to establish multi-center collaborations, generate large and diverse public datasets, and design prospective clinical trials. The integration of AI tools into ophthalmic software, the development of explainable interfaces for clinicians, and the clarification of regulatory pathways are essential next steps. With these advances, AI can evolve from a promising research tool to a reliable and impactful asset in the diagnosis and management of DED. 

## Figures and Tables

**Table 1:** DL-based classification of DED subtypes using videokeratography.

Study Ref.	Year	Purpose	Learning Model	Data Source	Development Strategy	Correlation with Experts	Results	Limitations
[41]	2023	DED subtype classification.	3D-CNN	- 243 eyes; (31 sADDE; 73 mADDE; 84 DWDE;55 IEDE).	- Training (146 cases); - Test (97 cases); - DA (training = 2628 cases); - TFOD labeling.	- Strong correlation with TFOD-based subtypes, with highest accuracy in sADDE (p < 0.001).	- Accuracy: 78.4%; - sADDE 92.3%; - mADDE 79.3%; - DWDE 75.8%; - IEDE 72.7%.	- Unequal class distribution; - High diagnostic complexity.

Abbreviations: DE = dry eye; sADDE = severe aqueous deficient dry eye; mADDE = mild-to-moderate aqueous deficient dry eye; DWDE = decreased wettability dry eye; IEDE = increased evaporation dry eye; DA = data augmentation; TFOD = tear film-oriented diagnosis.

**Table 2:** DL-based classification of DED using smartphone-acquired images.

Study Ref.	Year	Purpose	Learning Model	Data Source	Development Strategy	Correlation with Experts	Results	Limitations
[42]	2024	TMH via smartphone.	U-Net + MobileNetV2.	- 1021 images (734 patients).	- Segmentation; - Cropping; - Super-resolution; - TMH calculation from light reflection.	- Matched iris diameter and pixel metrics vs. manual.	- Dice coefficient: 98.68%; - Overall accuracy: 95.39%.	- Reflectivity may vary across devices.
[44]	2025	Grading conjunctival hyperemia.	DeepLabV3 + LR.	- 29,640 images (450 DED patients).	- Segment the conjunctiva; - Predict redness grade; - Data split: training 80%, validation 20%.	- MAE of 0.450, strong alignment with expert scoring.	- 93.0% of predictions within 1 unit of the expert mean grade.	- Limited feature space; - Trial-specific protocols.

Abbreviations: TMH = tear meniscus height; MAE = mean absolute error; LR = linear regression.

**Table 3:** DL-based analysis of tear film interferometry for DED assessment.

Study Ref.	Year	Purpose	Learning Model	Data Source	Development Strategy	Correlation with Experts	Results	Limitations
[46]	2022	Incomplete blinking detection.	U-Net CNN	- 1019 video frames (100 subjects).	- Resizing to 512 × 512; - 80/10/10 split; - DA (flipping, rotation, translation, and scaling); - 100 epochs with early stopping.	IB frequency correlated with - TMH (R = −0.195); - NIBUT (R = −0.230); - TO (R = 0.311); - CLGS (R = 0.364); - OSDI (R = 0.304); - All p < 0.05.	- Dice = 0.925; - IoU = 0.887; - Sensitivity = 0.994 for 30 FPS videos; - IB frequency higher in DED (p < 0.001).	- Slightly lower accuracy with 30 FPS; - Only 30 FPS and 8 FPS videos were tested.
[47]	2025	Automatic TMH measurement.	Mask R-CNN (ResNet-101)	- 1200 OSIs (100 external validation).	- 70/20/10 split; - 200 epochs.	- R^2^ = 0.92 vs. expert.	- IoU: 0.928; - AUC: 0.935.	- Limited sample size; - Same device/same center; - No other clinical factors.
[49]	2023	TMH measurement.	DeepLabv3 + ResNet50 + GoogleNet + FCN.	- 305 OSIs.	- CNN segmentation; - Region detection (CCPR); - TMH quantification with regression.	- r^2^ = 0.94 vs. manual.	- Dice = 0.884; - Sensitivity = 0.877.	- Moderate sample size; - Relies on static image quality.

Abbreviations: NIBUT = non-invasive tear film breakup time; TO = tear osmolarity; CLGS = conjunctival lissamine green staining; OSI = ocular surface image; OSDI = ocular surface disease index; IB = incomplete blinking; CCPR = central corneal projection ring; FPS = frames per second; IoU = intersection over union; AUC = area under curve.

**Table 4:** DL-based classification of DED using AS-OCT imaging.

Study Ref.	Year	Purpose	Learning Model	Data Source	Development Strategy	Correlation with Experts	Results	Limitations
[50]	2021	Compare DL to clinical tests (AS-OCT).	VGG19	- 27,180 AS-OCT Images (151 eyes).	- Exclude poor quality images; - 14,294 training; - 3574 validation; - 7020 test.	- Significant agreement with CFS, CLGS, Schirmer; - No correlation with TBUT, OSDI (p > 0.18).	- Accuracy 84.62%; - Sensitivity 86.36%; - Specificity 82.35%.	- No gold standard; - Same device and same center; - Limited dataset.

Abbreviations: TBUT = tear film breakup time; OSDI = ocular surface disease index; AS-OCT = anterior segment optical coherence tomography; CFS = corneal fluorescein staining; CLGS = conjunctival lissamine green staining.

**Table 5:** DL-based analysis of infrared meibography for DED and MGD assessment.

Study Ref.	Year	Purpose	Learning Model	Data Source	Development Strategy	Correlation with Experts	Results	Limitations
[51]	2025	Standardize MG absence detection.	3 CNN: (IQD, OFD, GAD)	- 143,476 meibography (15 sites); - 135,251 after cleaning.	-80/10/10 split. - Class balancing for IQD/OFD; - Data annotation.	- Cohen’s κ: 0.60–0.71.	IQD: - AUROC = 0.88; - Precision = 0.81. OFD: - AUROC = 0.91; - Precision = 0.88. GAD: - AUROC = 0.93; - Precision = 0.84.	- No external validation; - Limited interpretability.
[52]	2021	Assess MG morphology + clinical link.	Mini U-Net CNN.	- 120 Meibography images.	- Preprocess; - DA; - Data split (120 epochs).	- Correlated with clinical metrics; - Repeatability = 100%.	- IoU: 0.9077. - Processing time: 100 ms/image.	- Small sample size; - Minor accuracy gain but 10× more parameters.
[53]	2023	MG + tarsus segmentation.	- ResNet18; - U-Net + ResNet34;VAE-GAN.	- 1087 images (366 DED patients).	- 70/20/10 split; - Preprocessing: Cropping, bilateral filtering, max-min normalization. - DA: Color jittering, random lighting, horizontal flips, Gaussian noise.	- Strong agreement with experts (TS); MGS sens 75%.	TS: - AUC = 0.985; - Accuracy = 98.5%. MGS: - AUC = 0.938; - Accuracy = 93.7%.	- Single-device data; - No symptom link; - Moderate sensitivity.
[55]	2022	MG loss quantification.	R-CNN	- 1878 images (475 patients).	- 80/20 split; - Independent test (58 images); - Preprocessing.	-r = 0.976; - Mean difference = 3.55% (±2.12%).	- mAP > 92%; - VL <1.0.	- Single-center; - Small sample size; - No external validation.
[56]	2021	MG segmentation + assessment.	Inception ResNet + U-net	- 728 images.	- Data annotation; - Data preparation; - Design & training; - Model evaluation; - Training & validation (628); - Testing (100 images).	- Similar to manual p > 0.005.	- 100% repeatability; - Segmentation time = 1.33 s per image - Accuracy = 84%	-Device specific; -Partial segmentation success.
[57]	2022	Segment MG + eyelids, estimate meiboscore, remove reflections.	- ResNet34-based encoder-decoder; - Custom CNN; - GAN.	- 1000 images; - External Test (600 images).	- Annotation; - 200 epochs; - DA: Shear, horizontal flip (15%).	- 73.01% meiboscore agreement; - k = 0.20 − 0.71.	IQD: -AUROC = 0.88; Precision = 0.81. OFD: AUROC = 0.91; Precision = 0.88. GAD: AUROC = 0.93; Precision = 0.84.	- Internal validation only; - Device specific.
[58]	2024	Predict DED signs, symptoms, diagnosis from MG morphology.	Supervised segmentation + LR.	- 562 images (363 subjects).	- Data annotation; - Preprocessing; - Feature selection; - 5-fold CV.	- Compared to expert labels.	- Signs: Accuracy 72.6–99.1% - Symptoms: Accuracy 60.7–86.5% - Diagnoses: Accuracy 73.7–85.2%	- Lower accuracy for symptom; - Single-center dataset.
[59]	2025	Early DED detection & classification.	- UNet++; - ESAE; - SLSTM-STSA; - EQBFOA.	- 1000 MG images (320 patients).	Multi-stage pipeline: - UNet++ segmentation; - COFA; - Optimization with EQBFOA; - Classification with SLSTMSTSA.	- High agreement in classification.	- 96.34% accuracy (SL-FRST).	- Computationally intensive; - Requires real-time testing.
[61]	2020	Segment + quantify MG.	CNN19	- 400 Hand-held; - 400 Keratograph images.	- DA using ET and P; - Gland segmentation; - Metrics computation; - Training 600; - Testing 200.	The *p*-values p > 0.005 vs. ground-truth.	For diseased cases: - Nbr of glands =13.11; - Tortuosity = 1.29; - Width = 4.3; - Length = 40.31; - Gland-drop = 0.70.	- Limited sample size.

Abbreviations: MG = Meibomian gland; IQD = image quality detection; OFD = over-flip detection; GAD = gland absence detection; VAE-GAN = variational autoencoder–generative adversarial network; VL = validation loss; TS = tarsus segmentation; MGS = Meibomian gland segmentation; ESAE-ODNN = enhanced stacked auto-encoder assisted optimized deep neural network; SLSTM-STSA = sequence-to-sequence deep learning framework; EQBFOA = enhanced quantum bacterial foraging optimization algorithm; COFA = chaotic version of the optimal foraging algorithm; P = perturbation; ET = elastic transformation.

**Table 6:** DL-based analysis of in vivo confocal microscopy for DED and MGD assessment.

Study Ref.	Year	Purpose	Learning Model	Data Source	Development Strategy	Correlation with Experts	Results	Limitations
[62]	2021	MGD Detection.	ResNet 34	- 24,919 images (NG = 2896, NMGOG = 830, MGAG = 3585, MGAOG = 1745, MGOG = 3086, MGOOG = 488).	- Extract features; classify images; - Data split: 70/30; - 12,889 evaluation.	- High agreement with the experts.	- Accuracy 88.1–93.9%; - AUROCs >0.95.	- Single architecture; - South China data only.
[63]	2021	Classification of MGD types.	DenseNet169	- 8311 VLCMI images (Healthy = 2766 OMGD = 2744 AMGD = 2801).	- Exclude poor quality images; - Train DenseNet121, DenseNet169, DenseNet201; - Data split: 60/40.	- Expert accuracy 91%.	- DenseNet169: 97% OMGD, 99% AMGD and 98% healthy.	- Only 2 structural abnormalities of MG; - Single-center.
[64]	2020	Automated CNF segmentation/evaluation.	CNS-Net	- 691 IVCM images (104 patients).	- Training 70% (483); - Validation 10% (69); - Test 20% (139); - DA: Flipping, rotation, PCA noise.	- AUC: 0.96; - mAP: 94%; Bland–Altman: - CCC = 0.912; -Bias = −49.2 pixels.	- Sensitivity = 96%; - Specificity = 75%; - RDR = 16%.	- Inability to quantify nerve tortuosity/width; - Single-center.
[65]	2021	Compare GAN vs. U-Net for corneal sub-basal nerve segmentation.	- GAN; - U-Net.	- 510 IVCM images (85 subjects).	- Training 403 images (augmented); - Testing: 102 images.	- ICC (inter-rater): 0.97 (95% CI: 0.96–0.98).	- GAN: AUC = 0.944; - U-Net: AUC = 0.893.	- Small dataset; - Device-specific; - No external validation.
[66]	2021	Quantify CSNT & correlate with clinical parameters.	- IPACHI; - KNN.	- 1501 IVCM (49 patients).	- Preprocessing; - Local-phase-based enhancement; - IPACHI segmentation.	- OSDI (r = 0.418); - TBUT (r = −0.398); - High expert agreement.	- F value (220.699).	- Limited sample size; - Not tear composition data; - Not analyzing the whole corneal nerve plexus.
[67]	2022	CNF + DC segmentation & evaluation.	U-Net + Mask R-CNN	- 1219 IVCM images (China); - 754 IVCM images (Germany).	- Training: 10-fold CV; - DA: flips, rotation, gamma contrast, random crop.	CNFs:- Nerve number: 0.85; - Length: 0.87; - Branching points: 0.95; - Tortuosity: 0.88. DCs: - Cell number: 0.95; - Size: 0.92.	CNF Model: - Sensitivity = 86.1%; - Specificity = 90.1%; DC Model: - Precision = 89.37%; - Recall = 94.43%; F1 score = 91.83%.	- Small cohorts; - Manual annotation variability is not fully addressed.

Abbreviations: VLCMI = in vivo laser confocal microscope; OMGD = obstructive Meibomian gland dysfunction; AMGD = atrophic Meibomian gland dysfunction; CNFs = corneal nerve fibers; CNS = corneal nerve segmentation; NG = normal group; NMGOG = normal with Meibomian gland opening group; MGAG = Meibomian gland atrophy group; MGAOG = Meibomian gland atrophy with obstruction group; MGOG = Meibomian gland obstruction group; MGOOG = Meibomian gland obstruction with opening group; CSNT = corneal sub-basal nerve tortuosity; ICC = interclass correlation coefficient; DCs = dendritic cells; KNN = K-nearest neighbors; RDR = relative deviation ratio; IPACHI = infinite perimeter active contour with hybrid region information.

**Table 7:** DL-based analysis of slit-lamp photography for DED diagnosis.

Study Ref.	Year	Purpose	Learning Model	Data Source	Development Strategy	Correlation with Experts	Results	Limitations
[68]	2023	Corneal staining evaluation.	SVM	- 421 slit-lamp images.	- Image preprocessing; - ROI detection; - Segmentation; - Feature extraction; - Feature selection; - Models tested: DT, BT, NB, KNN, RF.	- ICC = 0.789.	- Accuracy: 82.67%; - AUC: 96.59%.	- Single-center with moderate dataset size; - Large- or low-contrast staining patches.
[69]	2022	DED severity grading.	ImageJ	- 50 fluorescein images (slit lamp).	- Preprocessing; - Analysis; - Count the number of particles; - OGS grading; - 30 test images.	- Moderate intrarater (K = 0.426).	- Sr = 0.91 vs. true Oxford grade.	- Limited sample size.
[70]	2024	Automated DED severity grading.	- U-Net + VGG16.	- 1400 images (1100 DED patients); - 94 External validation.	- 5-fold CV; - Grid-based corneal division; - Trained on 200 labeled images.	- Internal validation: r = 0.868; - External validation: r = 0.863; - Inter-rater agreement: r = 0.920;	- Accuracy = 89%; - Sensitivity = 82%; - Specificity = 96%; - AUC = 0.97.	- Some zone occlusion; - No yellow filter; - Risk of FN in low contrast cases.
[71]	2024	Corneal staining grading.	ResNet50 (FKD-CSS)	- 3847 images (37 hospitals).	- ROI extraction; - Lesion-aware teacher; - Interpretable regression.	- Pearson r = 0.84–0.90 externally, outperforming experts (AUC ≤0.761);	- AUC = 0.881	- Moderate severity grading; - Limited to Chinese (Asian) population.
[72]	2023	Estimate TFBUT, DED.	Swin Transformer.	- 16,440 frames (158 eyes).	- T raining: 12,011; - Validation: 2830; - Test: 1599 frames; - DA; - Resize and crop.	- Spearman r = 0.791 with EMR TFBUT.	- Sensitivity: 0.778; - Specificity: 0.857; - AUC: 0.813; - PPV: 0.875; - NPV: 0.750.	- Small and imbalanced dataset; - Model based only on the Japanese population.
[73]	2024	Estimate TBUT.	DTSN (InceptionV3, EfficientNetB3NS).	- 67 videos (France).	- Frame classification + temporal smoothing.	- Pearson r = 0.81 vs. ophthalmologist.	- AUC = 0.870.	- Single centered; - Limited diversity.
[74]	2020	SPK Grading.	CNN	- 101 slit lamp images.	- Data collection; - Ophthalmologist grading; Manual selection of the ROI; - Model training; - DA; - CNN-SPK detection.	- CNN-SPK vs. clinical grading (r = 0.85, p < 0.05).	- Sensitivity = 0.94; - Specificity = 0.79; - Accuracy = 97%.	- One eye per patient; - Limited sample size.
[75]	2024	Detection + monitoring of DED.	U-Net + YOLOv5.	- 2440 slit-lamp; - 1200 meibography.	Dual-mode RGB/IR CNNs + postprocessing.	Model matched expert MG evaluation.	- Accuracy 98.7%; - F1 up to 0.980.	- Clinical validation needed for long-term deployment.

Abbreviations: LC = Linear correlation; CNFL = corneal nerve fiber length; CI = confidence interval; SVM = support vector machine; ROI = region of interest; DT = decision tree; BT = Boosting Tree; NB = naive Bayes; RF = random forest; EMR = electronic medical records; PPV = positive predictive value; NPV = negative predictive value; PEE = punctate epithelial erosion; OGS = oxford grading schema; SPK = superficial punctate keratitis; DTSN = Dual-Task Siamese Network; FCN = fully convolutional network; OSD = ocular surface disease; RGB = red green blue; FKD-CSS = fine-grained knowledge distillation corneal staining score.

## Data Availability

No new datasets were generated for this review. All data supporting the findings of this study are derived from previously published sources, which are cited in the manuscript and are accessible through their respective publishers.
